# Incidence and risk factors of acute bilirubin encephalopathy in neonates with hyperbilirubinemia presenting at secondary care hospital

**DOI:** 10.12669/pjms.39.2.5996

**Published:** 2023

**Authors:** Saba Rauf, Bushra Salah ud Din, Ghanwa Abbas, Zeeshan Nawaz

**Affiliations:** 1 Dr. Saba Rauf, Women Medical Officer (WMO), THQ Hospital Pindigheb, Punjab Pakistan; 2 Dr. Bushra Salah ud Din, Women Medical Officer (WMO), THQ Hospital Pindigheb, Punjab Pakistan; 3 Dr. Ghanwa Abbas, Women Medical Officer (WMO), THQ Hospital Pindigheb, Punjab Pakistan; 4 Dr. Zeeshan Nawaz, Medical Officer (MO), THQ Hospital Pindigheb, Punjab Pakistan

**Keywords:** Frequency, Factors, Acute bilirubin encephalopathy, Newborn, Hyperbilirubinemia

## Abstract

**Background & Objectives:**

In neonates, hyperbilirubinemia is the most common problem. Acute bilirubin encephalopathy is serious problem in less developed nations and their incidence varies with geographical location. Our objective was to find out the incidence and risk factors of acute bilirubin encephalopathy in neonates with hyperbilirubinemia

**Methods:**

This was a prospective cross sectional study carried out at the department of pediatrics, THQ Hospital, Pindigheb in Punjab from October 2020 to October 2021. The inclusion criterion for our study was all the neonates of both the gender diagnosed with hyperbilirubinemia. Blood samples were collected from all the neonates and were sent to hospital laboratory for total serum bilirubin (TSB) measurement. The risk factors like delivery place, Rh/ABO incompatibility and preterm delivery were recorded. SPSS version 23 was used to input and analyze all of the data.

**Result:**

In this study, a total of 350 neonates were included. The prevalence of acute bilirubin encephalopathy in our study was 16% (n=56). Seven (12.5%) neonates with acute bilirubin encephalopathy were preterm which was the major significant risk factor for acute bilirubin encephalopathy in hyperbilirubinemia neonates (p<0.05). The other risk factors such as hospital delivery, birth weight, Rh and ABO incompatibility were also observed but were non-significant (p>0.05).

**Conclusion:**

Our study observed 16% prevalence of acute bilirubin encephalopathy in neonates with hyperbilirubinemia. The preterm birth was a significant risk factor associated with the acute bilirubin encephalopathy in neonates with hyperbilirubinemia.

## INTRODUCTION

In neonates, hyperbilirubinemia is the most common problem and majority (above 80%) of the cases occurs in preterm neonates.^1^ Acute bilirubin encephalopathy (ABE) in newborns is one of the most serious complications and may result in irreversible central nervous system damage.^2^ Although the prevalence of ABE in industrialized nations has declined currently, still it occurs in 0.4 to 2.7 instances/100,000 newborns,^3^ with a higher prevalence in Africa, Asia and Middle East.^4^

In principle, if we can anticipate and act early enough, we can completely prevent this disorder. ABE has been linked to an increase in total serum bilirubin (TSB) level and to stratify the severity of hyperbilirubinemia, the National Institute of Health and Clinical Development (NIHCD) has classified is as extremely severe hyperbilirubinemia (EHB) if TSB is less than 25 mg/dl.^5^,^6^

Considering the manifestations of ABE, the lethargy, loss of Moro reflexes and poor feeding are the early signs whereas severe weakness, shrill cry, respiratory distress, convulsions, poor neonatal reflexes and opisthotonus posturing are late features of ABE. The majority of babies will full developed neurological signs may either die or remain with severe disabilities like chore form movements and spastic diplagia or monoplegia. 7

Many risk factors are associated with ABE like low birth weight, preterm birth, sepsis, blood group incompatibilities and G6PD deficiency.^8^ Based on literature search, limited data is avaiabe about ABE in Pakistan. Therefore this study was planned to determine the frequency and risk factors of acute bilirubin encephalopathy in neonates with hyperbilirubinemia at our center.

## METHODS

This was a prospective cross sectional study conducted at the department of pediatrics, THQ Hospital, Pindigheb in Punjab province of Pakistan. The study duration was one year from October 2020 to October 2021. This was approved by the hospital research and ethical committee (27/THQPG/2021). An informed consent was taken from all the guardian of the neonates. All the neonates of either gender who were diagnosed with hyperbilirubinemia were included in our study while neonates with incomplete information and those whose parents did not consent to participate in the study were excluded. Acute bilirubin encephalopathy was categorized into Mild and Moderate -Severe ABE based on clinical presentation. Mild ABE was defined as neonates with poor feeding, hypotonia/ hypertonia, lethargy and high pitch cry while moderate to Severe ABE was defined as neonates with seizures, flaccidity or rigidity, upward gaze paralysis, apnea and opisthotonus.

Blood samples collected from all the neonates were sent to hospital laboratory for total serum bilirubin (TSB), CBC, Reticulocyte count, blood group, blood cultures, G6PD level.

ABE was diagnosed on the basis of hemolytic anemia in neonates. ABE was confirmed if the level of TSB was ≥20 mg/dl and hematocrit (HCT) was ≤35%. Once ABE was diagnosed then neonates were divided into groups based on level of TSB. Neonates were labeled as at threshold of intervention, if the TSB level was 20-29.9 mg/dl while neonates were labeled as at high risk of development of acute bilirubin encephalopathy if the TSB level was ≥30 mg/dl.

Data was collected including gender, incidence of ABE in hyperbilirubinemia, severity of ABE and the risk factors like delivery place, Rh factor incompatibility, ABO incompatibility and preterm delivery. Mortality associated with ABE was also documented. SPSS version 23 was used to analyze the data. Mean and standard deviation were documented for continuous variables, while categorical variables were calculated as percentages and proportions. For comparing of risk factors, Chi-square test was used. A *p-value* of <0.05 was taken as significant.

## RESULTS

In this study a total of 350 neonates were included. There were 210 (60%) males while 140 (40%) neonates were female. We diagnosed ABE in 56 neonates among 350 with hyperbilirubinemia accounting for 16%.

According to the severity of ABE, 25 (7.14%) neonates had mild acute bilirubin encephalopathy while 31 (8.86%) had moderate to severe ABE. In 26 (48%) neonates with ABE, the level of TSB was in range from 20-29.9 mg/dL, in 6 (10%) newborns with ABE, TSB level was greater than 30 mg/dl while in 24 (42.86%) newborns with ABE, TSB level was less than 20mg/dL. The overall mortality rate in our study was 5.4% (n=3) in neonates with ABE. (Table-I)

**Table-I:** Major features and outcome of neonate with ABE.

Parameter	Category	Frequency	Percentage
Severity of ABE	Mild ABE	25	7.14%
	Moderate to severe	31	8.86%
TSB level in neonates with ABE	20-29.9 mg/dl,	26	48%
	>30 mg/dl	6	10%
	<20.	24	42.86%
Outcomes in neonates with ABE	Survived	53	94.64%
	Mortality	3	5.4%

In our study, seven (12.5%) neonates with ABE were preterm. The risk factor, preterm birth was significantly associated with the risk of ABE in hyperbilirubinemia neonates (p<0.05). There were 18 (32.14%) neonates with ABE had birth in hospital but this was not significantly associated with the risk of ABE (p>0.05). Birth weight was observed to have no significant association with the risk of ABE (p>0.05). In our study, Rh factor incompatibility and ABO incompatibility were observed in 2 (3.57%) and 17 (30.36%) neonates with ABE. Both these factors were non-significantly associated with the risk of ABE (p>0.05) (Table-II).

**Table-II:** Risk factors associated with ABE in neonates with hyperbilirubinemia.

Risk factors		Neonates with ABE (n=56) N (%)	*P-value*
Preterm birth		7 (12.5%)	0.001
Birth place	Hospital	18 (32.14%)	0.091
	Outside Hospital	38 (67.86%)	
Birth weight	<1500 grams	3 (5.36%)	0.231
	1500-2499 grams	17 (30.36%)	
	>2500 grams	36 (64.29%)	
Hematologic reason for Hyperbilirubinemia	Rh factor incompatibility	2 (3.57%)	0.071
	ABO incompatibility	17 (30.36%)	

## DISCUSSION

Three hundred fifty neonates were included in this study. There were 210 (60%) male neonates while 140 (40%) neonates were female in our study. In accordance to our study, a previous study also reported male predominance and reported that hyperbilirubinemia in male neonates was 57%.^9^ Another study also reported higher incidence of hyperbilirubinemia in male neonates as compared to female.^10^

The incidence of ABE in our study was 56 (16%). A previous study reported comparable incidence of ABE to our study.^11^ According to the category of ABE, 25 (7.14%) neonates were observed with mild ABE while moderate to severe ABE was observed in 31 (8.86%) neonates. A previous study reported comparable results to our findings.^12^ In 26 (48%) neonates with ABE, the level of TSB was in range from 20-29.9 mg/dL, in 6 (10%) newborns with ABE, TSB level was greater than 30 mg/dl while in 24 (42.86%) newborns with ABE, TSB level was less than 20mg/dL in our study. TSB has been linked to an increased risk of ABE in certain studies. According to previous study the incidence of ABE is reduced in newborns with a TSB of less than 30 mg/dL.^13^ Whereas ABE has been observed in a previous study in 38.26% newborns with total bilirubin serum less than 20 mg/dL, many of these newborns had sepsis or had been pre-term.^7^

In our study, 7 (12.5%) neonates with ABE were preterm. The risk factor, preterm was significantly associated with the risk of ABE in hyperbilirubinemia neonates (p<0.05). Contrary to our study, a previous study done by Arnolda et al. in Myanmar reported that rate of preterm birth was higher in non-ABE neonate as compared to ABE neonate.^14^ According to an earlier study, preterm newborns have high risk to develop acute bilirubin encephalopathy and have high chance of long term neurologic problems as a result of ABE or as a result of unnecessary treatment.^15^ In our study 18 (32.14%) neonates with ABE had birth in hospital but this was not significantly associated with the risk of ABE (p>0.05). According to studies, ABE risk is influenced by the location of birth. Hyperbilirubinemia and associated therapeutic options are poorly understood in out-of-hospital delivery settings.^16^ Birth weight was observed to have no significant association with the risk of ABE (p>0.05). In our study, Rh factor incompatibility and ABO incompatibility were observed in 2 (3.57%) and 17 (30.36%) neonates with ABE. Both of these factors were non-significantly associated with the risk of ABE (p>0.05). In accordance to our findings a previous study reported comparable results.^17^

Another study done by Bhutani et al. reported the association of Rh incompatibility in the ABE incidence.^4^ According to research, low- and middle-income nations suffer the greatest impact of severe newborn hyperbilirubinemia, which is defined by a significantly greater ratio of illness, death, and neurological problems as compared to high-income countries 8. In addition, according to the research, about 4.3 percent of babies with jaundice are unable to sit or stand properly.^18^ In reality, infants are not diagnosed and treated appropriately quickly enough, resulting in widespread jaundice problems.^7^ Depending on the severity of the infant’s jaundice, it may develop to acute bilirubin encephalopathy (ABE).^19^ Survivors may suffer protracted neuro-developmental consequences such as brain paralysis, hearing loss, intellectual problems or major developmental delays.^20^ Clinical recommendations suggest early neonatal identification of individuals at risk of severe hyperbilirubinemia in order to treat the accompanying burden in a timely and effective manner.^21^

In neonates with hyperbilirubinemia, our research is one of the few studies done in Pakistan that has been able to identify the risk variables for the development of ABE. The identification of risk variables may aid in identifying high-risk hyperbilirubinemia neonates who are at risk for ABE. Our research study has some limitations, including the fact that it is a single-center study that only included newborns with hyperbilirubinemia. Furthermore, our hospital is located in a densely populated urban region. As a result, the actual incidence of ABE cannot be determined by this research as almost 70% of Pakistan’s population lives in rural regions, many births take place in inadequate health facilities or even at home. As a result, a multi-center research is needed to establish the precise prevalence of ABE in our community as well as the risk factors linked to ABE.

### Limitations of the study:

The primary drawback of the present research is the limited sample size and single center design. Additionally, our hospital is located in a heavily populated urban area. The real incidence of ABE cannot thus be determined by this investigation. Because over 70% of Pakistan’s population resides in rural regions, the majority of births in the country take place in either rudimentary healthcare facilities or at home. Therefore, it is necessary to perform a multi-center investigation to ascertain the precise prevalence of ABE in our area as well as the risk factors related to ABE.

## CONCLUSION

Our study concludes that the prevalence of ABE in this study was 16% in neonates with hyperbilirubinemia. The preterm birth was a significant risk factor associated with the risk of ABE in neonates with hyperbilirubinemia. In future, additional prospective observational studies from multiple centers will be required to give more clinical data regarding ABE in newborns with hyperbilirubinemia.

### Authors’ Contribution:

**SR, BSD, GA & ZN:** Conceived, designed and did statistical analysis & editing of manuscript, is responsible for integrity of research.

**BSD, GA & ZN:** Did data collection and manuscript writing.

**SR:** Did review and final approval of manuscript.
